# Studies of the Formation and Stability of Ezetimibe-Cyclodextrin Inclusion Complexes

**DOI:** 10.3390/ijms23010455

**Published:** 2021-12-31

**Authors:** Marta Biernacka, Tatsiana Ilyich, Ilya Zavodnik, Bartłomiej Pałecz, Artur Stepniak

**Affiliations:** 1 Unit of Biophysical Chemistry, Department of Physical Chemistry, Faculty of Chemistry, University of Lodz, Pomorska 163/165, 90-236 Lodz, Poland; marta.biernacka@chemia.uni.lodz.pl (M.B.); bartlomiej.palecz@chemia.uni.lodz.pl (B.P.); 2 Department of Biochemistry, Yanka Kupala State University of Grodno, BLK-50, 230030 Grodno, Belarus; tatyana-luchic@yandex.ru (T.I.); zavodnik_il@mail.ru (I.Z.)

**Keywords:** cyclodextrins, formation constant, isothermal titration calorimetry, differential scanning calorimetry, FT-IR, HNMR, mitochondrial permeability transition

## Abstract

In the presented studies, the interactions between ezetimibe (EZE) and selected cyclodextrins were investigated. α-Cyclodextrin (αCD), β-cyclodextrin (βCD) and its modified derivatives, hydroxypropyl-β-cyclodextrin (HPβCD) and sulfobutylether-β-cyclodextrin (SBEβCD), were selected for the research. Measurements were carried out using calorimetric and spectroscopic methods. Additionally, the Hirshfeld surface and biochemical analysis were achieved. As a result of the study, the inclusion complexes with 1:1 stoichiometry were obtained. The most stable are the complexes of β-cyclodextrin and its derivatives. The comparison of βCD with its derivatives shows that the modifications have an affect on the formation of more durable and stable complexes.

## 1. Introduction

**Figure 1:**
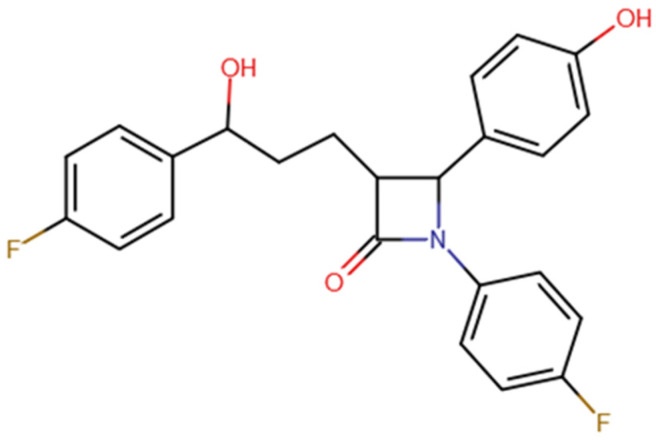
Structure of the ezetimibe molecule.

In recent years, organofluorine compounds have acquired a great interest in medicine and pharmacy. Due to the presence of a C-F bond with a remarkably high energy (≈513 kJ mol^−1^) [1], these compounds are characterized by a high thermal and chemical stability. As a result, they are highly stable in the environment [2,3]. Ezetimibe (1-(4-fluorophenyl)-3(R)-[3-(4-fluorophenyl)-3(S)-(4-hydroxyphenyl)-2-azetidinone) (Figure 1) is an organic compound that dissolves very well in all kinds of organic solvents, e.g., ethanol, DMSO, DMF, but it is practically insoluble in water.

Ezetimibe is used to treat hypercholesterolemia and to reduce the level of lipids in the blood. Its action is based on the inhibition of the absorption of both, dietary and endogenous cholesterol, without affecting the absorption of fat-soluble nutrients [4]. Ezetimibe works by selectively blocking a protein (NPC1L1), which is responsible for the transport of cholesterol in the intestines. This leads to the inhibition of lipids absorption in the gastrointestinal tract, causing the reduction of cholesterol level in the blood [5]. The use of the drug reduces the concentration of low-density lipoprotein, which in common language is known as “bad” cholesterol (LDL), as well as triglycerides, without affecting the concentration of “good” cholesterol (HDL) [6]. However, due to the low solubility of ezetimibe in water, its bioavailability in the human body may be changeable and partly limited.

Cyclodextrins (CDs) are natural macromolecules made of glucose units linked by α-1,4-acetal bonds. There are three basic natural cyclodextrins (α, β, and γ). They differ from each other in the number of glucose molecules, which form the ring of cyclodextrin molecule. Accordingly, α-CD has six, β-CD has seven, and γ-CD has eight glucose units [7]. Natural cyclodextrins are made from starch. This process takes place by using the enzymatic properties of some families of bacteria [7,8]. From a chemical point of view, cyclodextrins are polysaccharides of natural origin. They do not show any toxic properties [9,10], in contrast to the dendrimers and cucurbiturils [11], which are also used as nano-transporters of active substances [12].

Due to the specific structure of cyclodextrins, they can easily be solved in water. Cyclodextrins have also a hydrophobic gap, inside of which apolar molecules can be easily included. These molecules show a hydrophobic character (like most of the organic compounds) which is similar to the gap in cyclodextrin [13,14].

Due to this structure, cyclodextrins found their place in supramolecular chemistry. They have the ability to enclose guest molecules inside them by creating inclusion complexes. The formation of such a complex consists of the creation of a guest-host system between the molecules, without the formation of any covalent bonds. Only weak intermolecular interactions, such as van der Waals interactions and hydrogen bonds, are involved in the complex formation.

Cyclodextrins, linked with ligands, can form supramolecular structures similar to pseudorotaxanes [15,16,17]. Pseudorotaxanes are two unrelated chemical entities. One of them has a linear structure and constitutes the rotaxane axis, while the other is a macrocycle.

The process of inclusion leads to the formation of complexes that are characterized by an increased water solubility in relation to the uncomplexed compound, without affecting its biological properties. It has been proved, that the inclusion of some compounds inside the cyclodextrin macromolecule may improve their physicochemical properties [18,19]. The formation of complexes may enhance the chemical and thermal stability of these compounds [20]. The molecule, included in the complex, is protected by the macromolecule against a harmful influence of varied external factors such as high temperature or UV radiation [20,21]. Cyclodextrins can be successfully used in complexing medicines [22,23] and other biologically active compounds [24]. Song et al. showed that cyclodextrins can also be used to remove organic micropollutants from water [25]. They are fully permeable through biological membranes. They can increase the bioavailability of medicines, facilitate their transport in the body and prevent the process of aggregation of drug molecules in organs. Moreover, it has been proved that cyclodextrins can reduce irritation and side effects of drugs [26]. Cyclodextrins and their metabolites are excreted by the kidneys along with the urine, therefore there is no risk of accumulating them in the patient’s body [27,28].

The aim of the research was to determine the impact of selected cyclodextrins on the thermodynamics of interactions between ezetimibe and macrocycles. Studies have been carried out to create and determine the stability of ezetimibe inclusion complexes. Spectrophotometric research were performed to confirm the results obtained with calorimetric methods and to determine the increase of ezetimibe solubility in water.

## 2. Results and Discussion

### 2.1. Isothermal Titration Calorimetry

**Figure 2:**
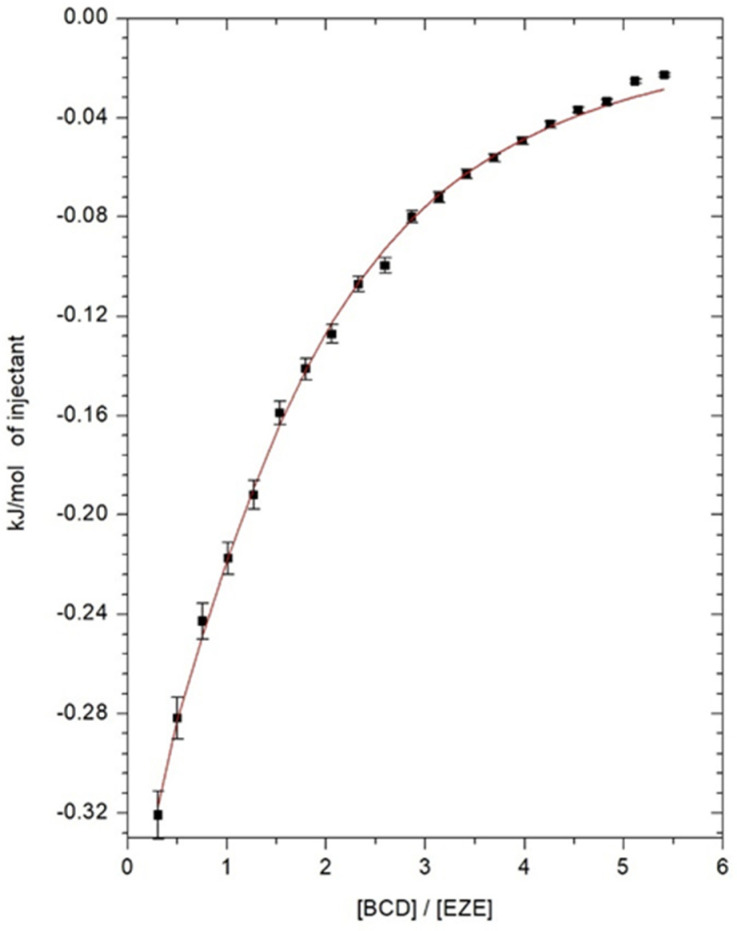
Direct effects of interaction between 0.5 mM ezetimibe and 14.2 mM α-cyclodextrin.

**Figure 3:**
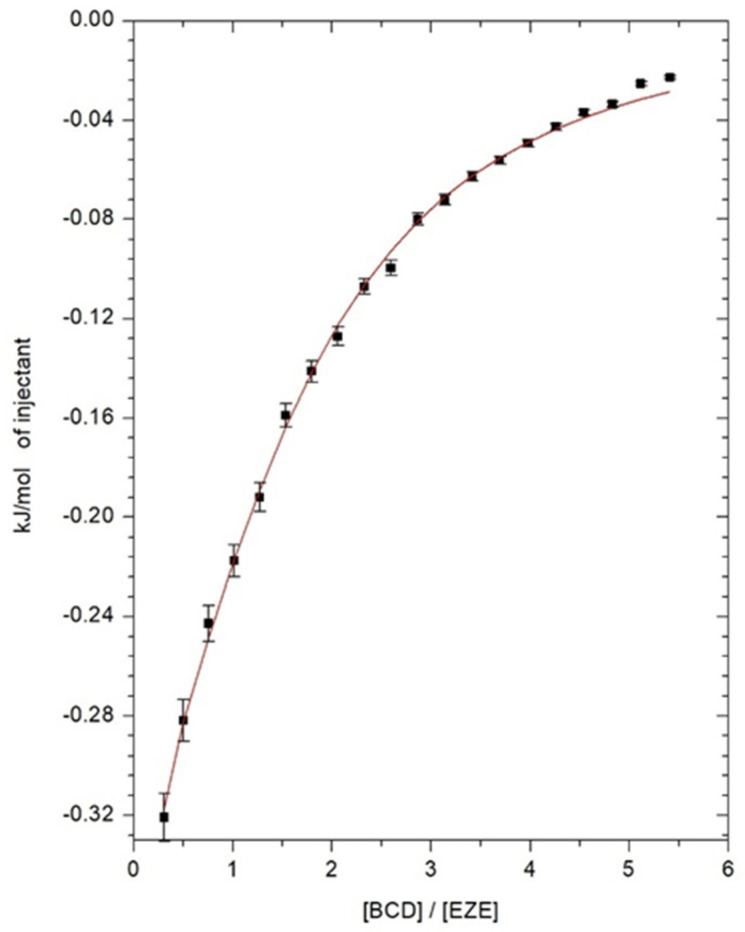
Direct effects of interaction between 0.5 mM ezetimibe and 14.2 mM β-cyclodextrin.

**Figure 4:**
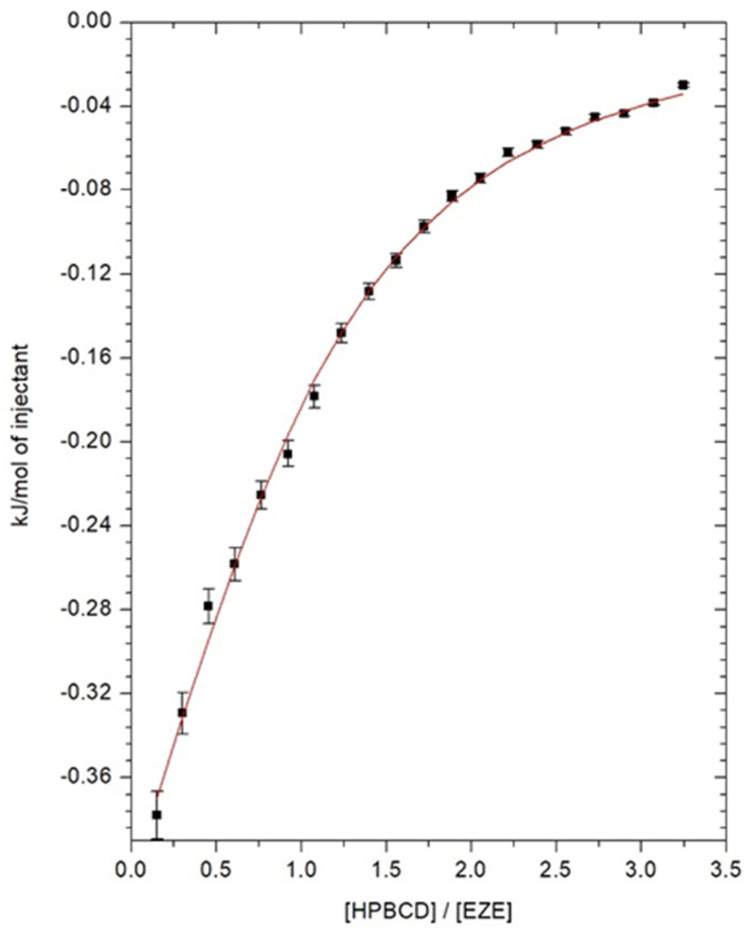
Direct effects of interaction between 0.5 mM ezetimibe and 8.52 mM HP-β-cyclodextrin.

To determine the thermodynamic parameters of the ezetimibe complexation process inside the cyclodextrin, isothermal calorimetry titration measurements were carried out. The thermal effects, describing the direct interaction of ezetimibe with αCD, βCD, HPβCD in the DMSO solutions as a function of the composition of the titrated solution, are shown in Figure 2, Figure 3 and Figure 4. The model of one active site was used for the mathematical description of the obtained thermograms. Based on this model, the stoichiometry of the created inclusion complexes (*N*), the formation constants of drug-cyclodextrin complex (*K*), molar enthalpy (Δ*H*), and entropy (Δ*S*) of the complexation processes, were determined and the free enthalpy (Δ*G*) was calculated. The values of thermodynamic parameters, describing the interaction of ezetimibe with αCD, βCD, and HPβCD in DMSO, are presented in Table 1. Due to the limited solubility of SBEβCD in the organic solvents, the ITC measurements were not performed for the ezetimibe-SBEβCD complex. The stoichiometric ratio (*N*), defining the number of ezetimibe molecules per one cyclodextrin macromolecule, in all analyzed cases, was close to one (Table 1). This indicates the formation of inclusion complexes with a stoichiometry of 1 (CD): 1 (EZE).

The obtained complex stability constants, for βCD and HPβCD complexes, have values *K* > 1000 dm^3^ mol^−1^. This confirms the formation of stable complexes between the ezetimibe and the molecules of these cyclodextrins. The enthalpy changes of the complexation processes of ezetimibe with all cyclodextrins are exothermic (Δ*H* < 0) (Table 1). The changes in entropy for all studied systems take on positive values (Δ*S* > 0) (Table 1), which proves the whole process of inclusion occurs spontaneously. Similarly, the changes in free enthalpies (Δ*G*) take on negative values, clearly indicating the spontaneity of the ongoing complexation process.

### 2.2. Differential Scanning Calorimetry

**Figure 5:**
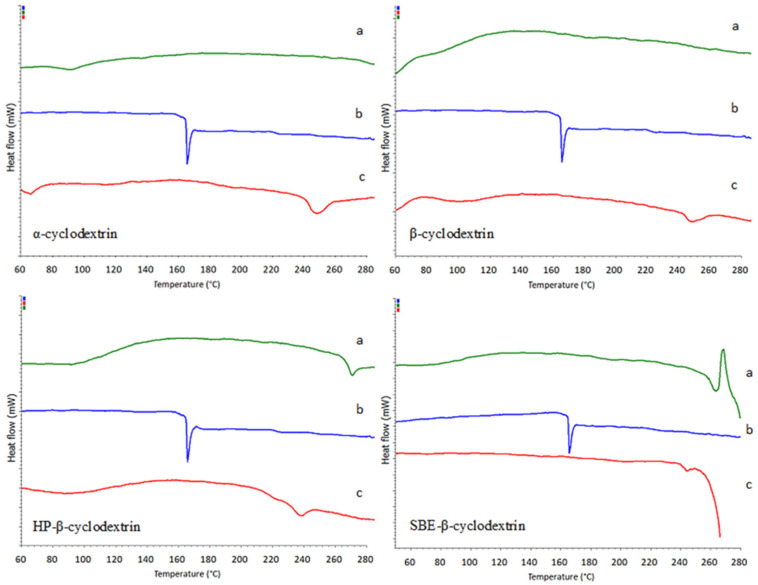
Differential scanning calorimetry thermograms of: cyclodextrin (**a**), ezetimibe (**b**) and ezetimibe/cyclodextrin complex 1:1 (**c**).

The measurements of pure ezetimibe, cyclodextrins (αCD, βCD, HPβCD, and SBEβCD) and the drug-cyclodextrin complexes were measured by differential scanning calorimetry. The obtained DSC thermograms are shown in Figure 5. The thermogram of pure ezetimibe shows a sharp endothermic peak at 165 °C which corresponds to the decomposition temperature of crystalline ezetimibe (lit 162 °C [29]). The thermograms of the pure cyclodextrins do not show any characteristic peaks in the analyzed temperature range, except for a minimal effect around 80–120 °C, caused by evaporation of crystal water, which is built into the cyclodextrins structure. In the case of thermograms of cyclodextrin-drug complexes, the sharp peak corresponding to the decomposition of ezetimibe disappears at the temperature characteristic for this compound. The only slight signal is visible around 240 °C The registered thermograms (Figure 5) indicate, that the decomposition temperature of ezetimibe, complexed with all cyclodextrins, shifts towards higher temperatures compared to the pure drug. The intensity of the endothermic peak is also reduced. The measurements showed that ezetimibe (included inside cyclodextrin macromolecules) is effectively protected against the influence of temperature. Thereby, the thermal stability of ezetimibe is increased.

### 2.3. Phase Solubility Study

**Figure 6:**
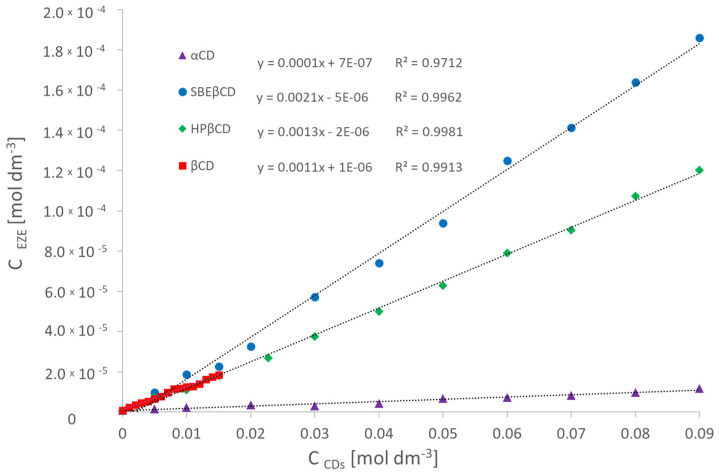
The dependence of solubility increases of ezetimibe in water on increasing concentration of αCD (▲), HPβCD (♦), SBEβCD (●) (1–90 mM), and βCD (■) (5–15 mM).

To establish the increase of ezetimibe water solubility, caused by the influence of rising concentration of cyclodextrins in the aqueous solution, UV-VIS spectrophotometry measurements were performed (Figure 6). Using the concept proposed by Higuchi and Connors [30], the solubility diagrams can be classified as the AL type. The observed increase of the drug solubility results from the formation of a drug-cyclodextrin inclusion complex with a stoichiometry of 1:1. The same stoichiometry for complexes βCD and HPβCD was obtained in studies performed by Mendhe et al. [31]. The obtained absorption spectra of the complexes show no maximum shifts caused by the bathochromic and hypsochromic effects in relation to the spectrum of the pure compound. Table 2 shows the increase of ezetimibe solubility in the presence of selected cyclodextrins. The stability constants of drug complexes with cyclodextrins were calculated based on Equation (1).

(1) $$K_{1:1}=Slope/S_{0}\left(1-Slope\right)$$

The presented measurements (Table 2) show that the addition of cyclodextrin to the aqueous drug solution causes a linear increase of ezetimibe solubility in water. Comparing the obtained results for α- and β-cyclodextrin with a concentration in the range of 0–15 mM (determining the maximum solubility of βCD) we observe a greater increase of the ezetimibe water solubility in the presence of βCD, than αCD. This is due to a better spatial fit of the larger βCD molecule (7 glucose units) to the structure of the ezetimibe molecule. Comparing the same range of concentration, the effect of modified β-cyclodextrin derivatives on the solubility of the drug in the water (Table 2), we notice a very similar growth trend.

Observing the influence of the modified beta-cyclodextrin derivatives on the ezetimibe water solubility, at their maximum concentrations, we notice, that in the case of complexing the drug with HPβCD, the solubility increases over 140 times, while the formation of the ezetimibe-SBEβCD complex increases the solubility of the drug by 200 times. The obtained values of stability constants of ezetimibe complexes with βCD, and its derivatives, determined by the Higuchi–Connors static method, have values of *K* > 1000 dm^3^ mol^−1^, which proves the formation of stable complexes.

### 2.4. FT-IR

**Figure 7:**
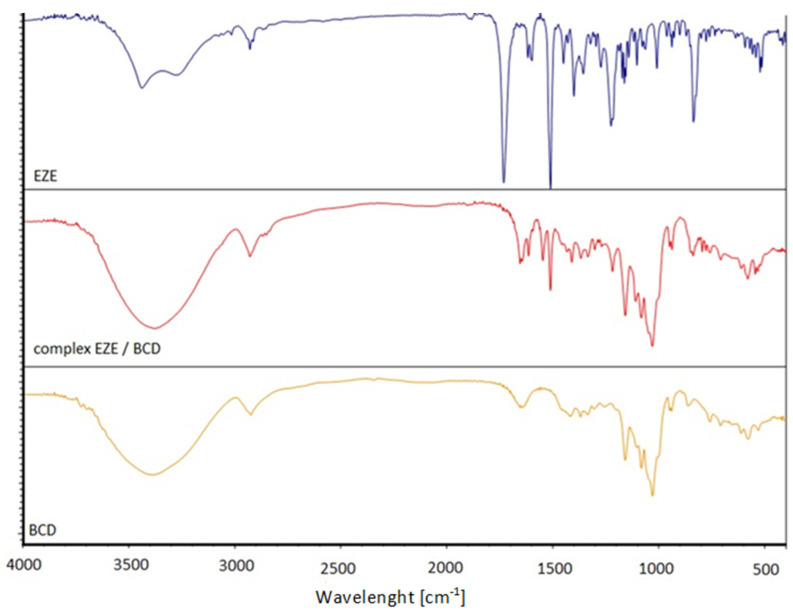
FTIR spectra of ezetimibe (EZE), β-cyclodextrin (βCD) and ezetimibe/β-cyclodextrin complex (complex EZE/βCD).

The following bands are visible on the FTIR spectra of all analyzed cyclodextrins: ~3400 cm^−1^ (O–H); ~2930 cm^−1^ (C–H); 1630–1640 cm^−1^ (H–O–H); ~1160 cm^−1^ (C–O), and ~1030 cm^−1^ (C–O–C). Similar results can be found in the literature [32]. The spectrum of ezetimibe corresponds with the literature data obtained experimentally [29,33]. The spectrum is characterized by the following bands: 3437.5 and 3275.2 cm^−1^ (O-H), 2928 cm^−1^ (C-H); 1730.6 cm^−1^ (C = O), 1509.7 cm^−1^ (C = C arom.), 1399.5 cm^−1^ (CN), 1224.0 cm^−1^ (CF), 834.5 cm^−1^ (p-substituted benzene ring). The FT-IR spectra of the complexes (Figure 7) are practically completely shielded by very wide and intense bands derived from cyclodextrins. Spectrum analysis, of all complexes, allows to observe several bands, that are characteristic of the ezetimibe. The bands at wavelengths of 1224.0 cm^−1^ for the C-F stretching vibration and bands at 1509.7 cm^−1^ for the C = C stretching vibration in the aromatic ring are visible, while the band at 1730.6 cm^−1^ (C = O) completely disappears. This disappearance of the band indicates that this area of the drug molecules is located inside of the cyclodextrin cavity and it is screened by the cyclodextrin molecule [34]. Similar results for βCD and HPβCD complexes were obtained by Patel et al. [35]. A clearly visible, intense band of 1509 cm^−1^ (C = C arom.) indicates that at least one of the aromatic rings in the ezetimibe structure remains exposed.

### 2.5. ^1^HNMR

Using the ^1^HNMR method, the following solutions were analyzed: ezetimibe (EZE), β-cyclodextrin (βCD), and their complex in the stoichiometry 1:1. The stoichiometry was based on spectroscopic and calorimetric titration measurements. The DMSO-6d was used as a solvent.

**Figure 8:**
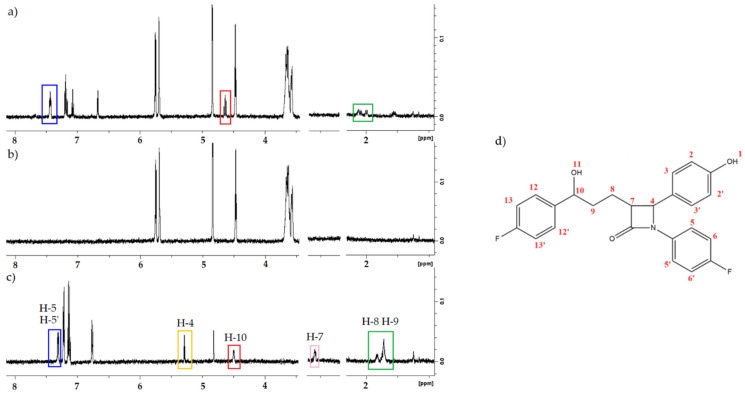
Spectra ^1^HNMR of (**a**) β-CD/EZE complex, (**b**) β-CD, (**c**) EZE; (**d**) protons assignment in the ezetimibe structure.

In the spectrum of ezetimibe, characteristic bands of chemical shifts can be observed. The bands in the complex spectrum correspond to the peaks of the following protons: H-5, H-8, H-9, H-10. Their positions are shifted towards higher values of chemical displacement (Figure 8). The signals from protons H-4 and H-7 disappear in the spectrum of the complex.

Changes in the chemical shifts seem to be caused by the formation of the β-cyclodextrin–ezetimibe complex.

### 2.6. MALDI TOF MS

**Figure 9:**
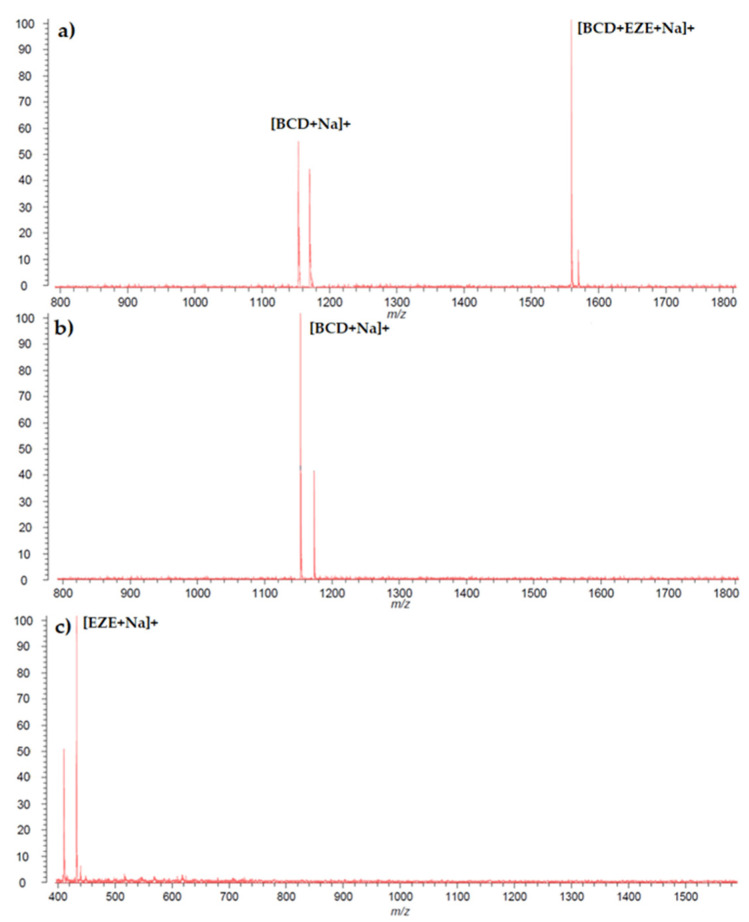
MALDI TOF mass spectrum of the (**a**) complex of ezetimibe and β-CD, (**b**) β-CD, and (**c**) ezetimibe.

The spectra obtained for ezetimibe, β-cyclodextrin, and their complex are presented in Figure 9. Particular peaks in spectra can be interpreted as follows: (c) m/z 432.6 as [EZE + Na]^+^; (b) m/z 1157.8 as [BCD + Na]^+^, (a) m/z 1157.8 as [BCD + Na]^+^ and m/z 1567.4 as [BCD + EZE + Na]^+^. The presence of the additional peak on the complex spectrum indicates the formation of the ezetimibe and β-cyclodextrin complex.

### 2.7. Hirshfeld Surface Analysis

**Figure 10:**
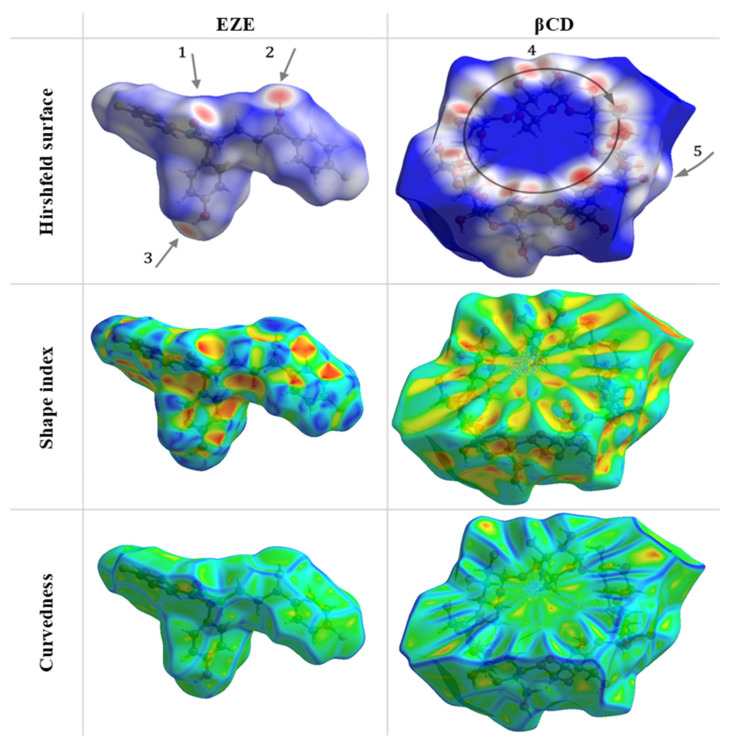
Hirshfeld surfaces mapped with d *_norm_*, shape index, and curves for ezetimibe and βCD. For the ezetimibe Hirshfeld surface, the water molecule was omitted for clarity. Arrows 1–5 indicate potential regions for hydrogen bonding.

To obtain a deeper insight into the intermolecular interactions of ezetimibe and βCD, the Hirshfeld surface approach was used exploiting the CrystalExplorer17 software [36]. This method visualizes the overall characteristic features of the intermolecular interactions and rises its popularity. Based on the results of CSD search we obtained the geometric parameters of ezetimibe molecule (QATNEF) [37] and cyclodextrin (WEWTOJ) [38]. The Hirshfeld surfaces of the ezetimibe and βCD are illustrated in Figure 10 showing the surfaces mapped over d *_norm_* (−1.2 to 1.4 Å), shape index (−1.0 to 1.0 Å), and curvedness (−4.0 to 0.4 Å).

**Figure 11:**
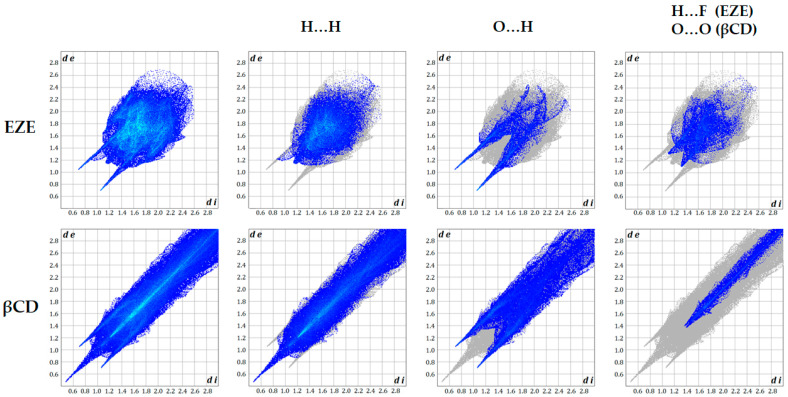
2D-fingerprint plots for ezetimibe and βCD.

The solid-state structure of ezetimibe includes a combination of O-H…O, C-H…O, and C-H…F interactions. The C-H…F interactions could be described as so-called “terminal” and probably do not interact with βCD. They are responsible for creating the chain between the molecules in the crystal packing. Other interactions due to their location in the molecular structure could be engaged in the interaction with βCD. The oxygen atoms from the hydroxyl groups act as donors for hydrogen bonds, while the carbonyl-oxygen atom acts as an acceptor. In the Hirshfeld surface these interactions are marked as 1, 2, and 3 and represented as two sharp spikes in the 2D fingerprint plots (Figure 10 and Figure 11) in the region 1.78 Å < (d*_e_* + d*_i_*) < 1.9 Å.

The molecule of βCD, due to a great number of potential donors and acceptors, indicates a net of hydrogen bonds which could be distinguish for two kind: O-H…O hydrogen bonds responsible for creating a stacks on the top edge of cyclodextrin molecule and C-H…O hydrogen bonds on the side edges of cyclodextrin molecule which connect “rings” of βCD molecules forming the plane of βCD rings parallel to (001) plane. The bright red spot labelled 4 is the O-H…O interaction with d*_i_* + d*_e_* equivalent to 1.7 Å, while 5 is the C-H…O interaction with the d*_i_* + d*_e_* equals 2.4 Å. The hydrophobic interior on the Hirshfeld surface is represented as the intensive blue color inside the βCD and pointed out that d norm value is longer than van der Waals separations.

**Figure 12:**
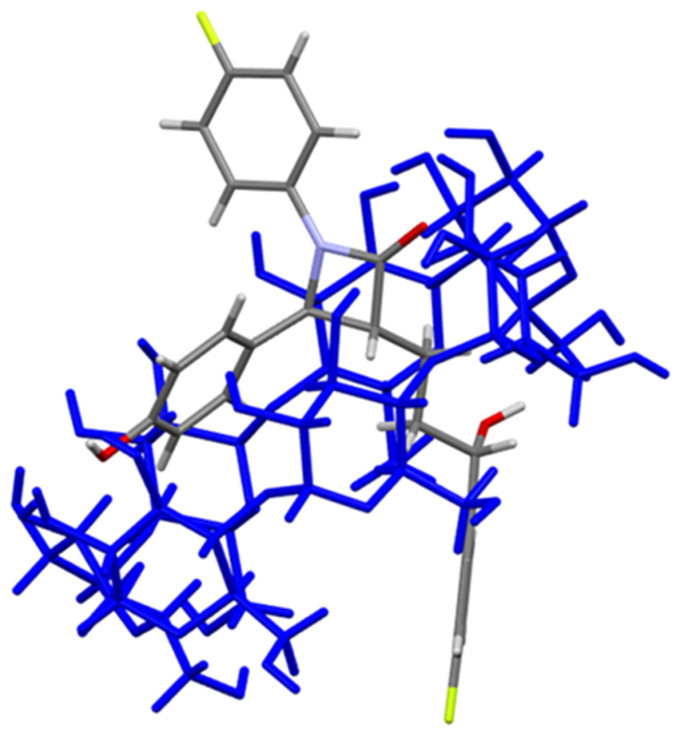
Proposed structure of the EZE-βCD complex.

Visualization of the Hirshfeld surface features, in the case of βCD, confirms the existence of the hydrophobic cavity, where close contacts are not possible (or greater then sum of van der Waals radii). In the case of ezetimibe Hirshfeld surface characteristic color coding suggests the regions 1, 2, and 3 as potential acceptor (1) and donor (2,3) of O-H…O interactions. Atom O1 would be a potential hydrogen bond acceptor for oxygens from the edges of the βCD molecule. While atoms O2 and O3 would be hydrogen bond donors for carbonyl oxygens (as acceptor) in the βCD molecule. These possible interactions may be responsible for the stabilization of the inclusion ezetimibe-βCD complex. Based on Hirshfeld surface studies, carried out using the crystallographic data, we proposed the way in which the ezetimibe molecule is located inside the cyclodextrin molecule (Figure 12).

### 2.8. Biochemical Analysis

**Figure 13:**
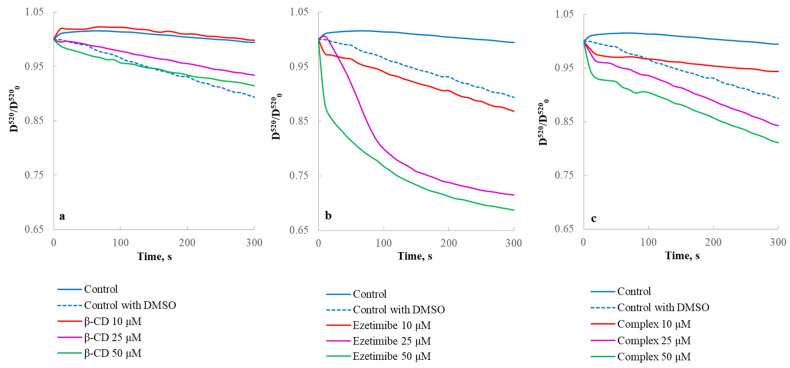
Effect of βCD/ezetimibe/complex ezetimibe-βCD on the process of mitochondrial permeability transition pores formation: (**a**) representative curves of mitochondrial swelling in the presence of βCD; (**b**) representative curves of mitochondrial swelling in the presence of ezetimibe; (**c**) representative curves of mitochondrial swelling in the presence of complex ezetimibe-βCD. Mitochondria (0.5 mg of protein/mL) were added into EDTA-containing medium: 0.24 M sucrose, 0.01 M Tris-HCl, 0.001 M EDTA, pH 7.2, at 25 °C.

The research shows the effect of βCD/ezetimibe/complex ezetimibe-βCD (10–50 µM) on the process of mitochondrial permeability transition (MPT) pores formation and the stability of mitochondrial membrane potential. Figure 13 shows representative curves of mitochondrial swelling (MPT), in the absence and in the presence of βCD/ezetimibe/complex ezetimibe-βCD (10–50 µM) (the mitochondrial isolation medium contained 0.001 M EDTA).

β-CD (10–50 µM) alone did not induce any significance mitochondrial swelling (Figure 13a). Ezetimibe (10–50 µM) dose-dependently increased the rate of swelling of isolated rat liver mitochondria (Figure 13b) in EDTA-containing media. The complex ezetimibe-βCD (10–50 µM) was less effective in increasing the rate of mitochondrial swelling in comparing with ezetimibe (Figure 13c).

**Figure 14:**
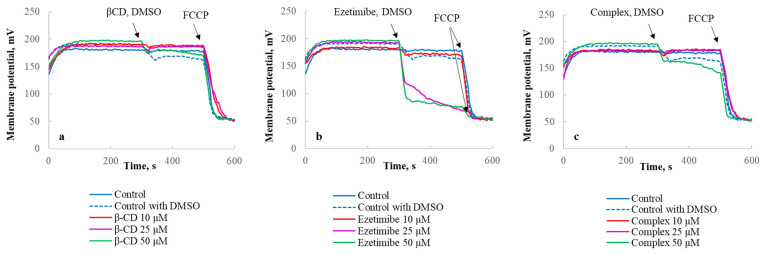
Representative traces of time-dependences of β-CD (**a**)/ezetimibe (**b**)/complex ezetimibe-β-CD (**c**)—induced dissipation of mitochondrial membrane potential. The arrows indicate additions of DMSO/β-CD/ezetimibe/complex ezetimibe-β-CD/FCCP to mitochondria suspension (0.3 mg of protein/mL) in the medium: 0.05 M sucrose, 0.125 M KCl, 0.01 M Tris–HCl, 0.0025 M KH_2_PO_4_, 0.005 M MgSO_4_, and 0.001 M EDTA, pH 7.5, at 27 °C. The mitochondrial membrane potential was detected using the fluorescent dye safranin O (8 µM) at λex/λem 495/586 nm at 27 °C and 5 mM succinate as substrates.

Similarly, βCD (10–50 µM) alone did not significantly change of the mitochondrial membrane potential value (Figure 14a). Ezetimibe (10–50 µM) caused a rapid dose-dependent dissipation of the mitochondrial membrane potential in a medium containing 0.001 M EDTA (Figure 14b). The complex ezetimibe-βCD (10–50 µM) causes a slight drop in the membrane potential (Figure 14c).

Since ezetimibe is a lipophilic agent, it is permeable to membranes [39]. In our experiments, ezetimibe induces mitochondrial swelling and membrane potential dissipation. The ezetimibe-βCD complex had less pronounced effects on the mitochondrial membrane parameters, which can be explained by the higher hydrophilicity of the ezetimibe-βCD complex compared to the initial substance alone.

It is well known that oxidative stress, mitochondrial dysfunction, and inflammation are related to cardiovascular diseases and atherogenesis [40]. Earlier, Hernandez-Mijares and coauthors showed that four weeks of monotherapy with ezetimibe or ezetimibe plus simvastatin significantly increased mitochondrial oxygen consumption, membrane potential, and glutathione content, and decreased levels of reactive oxygen species (ROS) production, thereby improving mitochondrial function in polymorphonuclear cells of hyperlipidemic patients [41].

For evaluating the possible effects of ezetimibe complexation with cyclodextrin on the biochemical activity of hypolipidemic drug we studied the changes of the mitochondrial functional state in the presence of ezetimibe/ezetimibe complex.

Mitochondria are dynamic and plastic organelles, taking part in a number of crucial metabolic processes, ATP synthesis, energy metabolism, the tricarboxylic acid cycle and β-oxidation of fatty acids, calcium storage, and signaling [42,43,44]. The MPT pore is a non-selective calcium and cyclosporine A (CsA)—sensitive high conductance channel that allows diffusion of solutes (<1500 Da) across the mitochondrial membrane and plays a crucial role in cell life and death [45,46,47].

Using isolated rat liver mitochondria, we showed the induction of the MPT pore formation by ezetimibe/complex expose in EDTA-containing media. We suggested that ezetimibe/complex induced pore formation in Ca^2+^—independent manner due to direct interactions with membrane proteins participating in pore formation. Another mechanism is protonophoric activity of the drug/complex. MPT pore formation was accompanied by membrane potential dissipation by ezetimibe/complex.

## 3. Materials and Methods

### 3.1. Materials

The materials used in this study were as follows: α-cyclodextrin (purity > 98%), β-cyclodextrin (>98%), HP-β-cyclodextrin (99%) (TCI), sulfobutylether-β-cyclodextrin (>95%) (Cyclolab, Budapest, Hungary) and ezetimibe (>98%) (Sigma Aldrich, St. Louis, MI, USA). The analytical grade organic solvents (ethanol and DMSO) were purchased from Sigma Aldrich. The substances used for the tests were dried in a vacuum dryer at 60 °C. The water used for calorimetric and spectrophotometric measurements of UV-VIS was triple distilled and degassed. For biochemical analysis, all solutions (β-CD/ezetimibe/complex ezetimibe-β-CD) were made with DMSO.

Cyclodextrins complexes in solid-state (for DSC, FTIR, ^1^HNMR, MS, and biological studies) were prepared by the co-evaporation method. Ezetimibe was dissolved in the ethanol, while cyclodextrins were dissolved in the triple distilled water. The ethanolic solution of ezetimibe had been gradually adding dropwise into the aqueous cyclodextrins solutions. The solutions prepared in this way were heated at 40 °C and mixed on a magnetic stirrer for about 48 h. Then, the solvent was completely evaporated from the solutions of the complexes (72 h at 60 °C).

### 3.2. Methods

#### 3.2.1. Isothermal Titration Calorimetry

**Figure 15:**
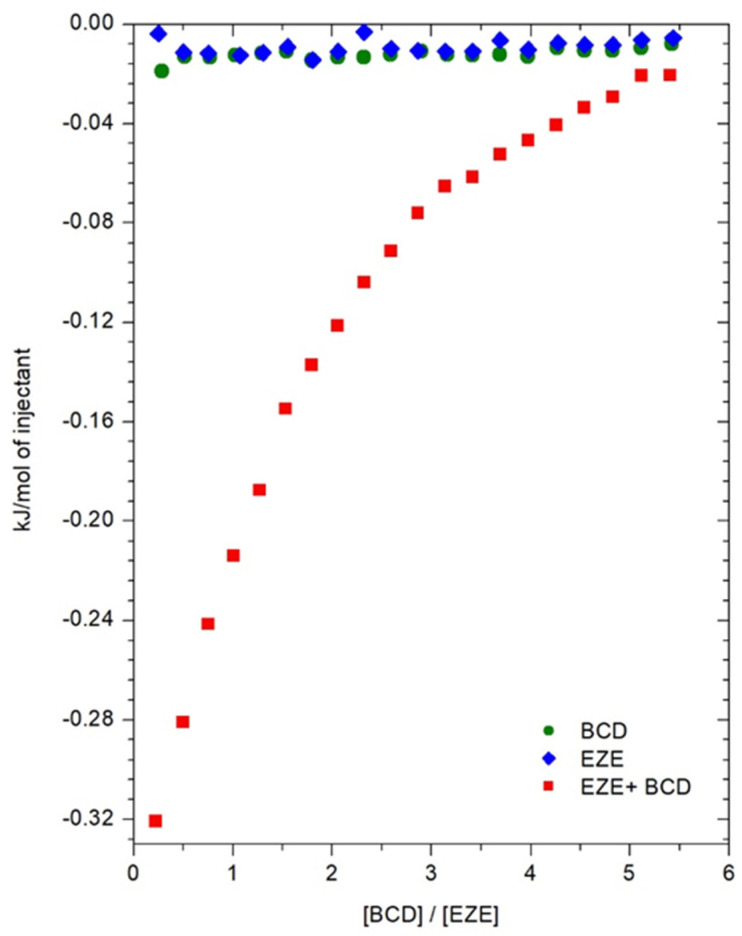
Thermograms describing energetic effects during the titration of a solution of ezetimibe with a solution of β-cyclodextrin (■), dilution of ezetimibe (♦), and dilution of β-cyclodextrin (●).

All isothermal calorimetric titration measurements were carried out at 25 °C using a Microcal VP-ITC microcalorimeter. Due to the low solubility of ezetimibe in water, measurements were performed in DMSO. The ezetimibe solution, with a concentration of 0.5 mM, was placed in the measuring cell. The cell volume was 1.42 mL. The cyclodextrin solution, with the concentrations as follows: (αCD—14.2 mM; βCD—14.2 mM, HPβCD—8.52 mM), had been titrating into the cell using the automatic syringe, which also worked as a stirrer, providing homogenous mixing of the titrated solution. The reference cell was filled with pure solvent (DMSO). Each measurement consisted of 20 injections, and each injection had a volume of 12.5 μL. The duration of each injection was 25 s, performed at 600 s intervals, at a stirrer (syringe) speed of 351 rpm. To eliminate the dilution effects, titrations of the drug solution with a pure solvent were performed, and all the cyclodextrins solutions were diluted with a pure solvent (DMSO) (Figure 15).

#### 3.2.2. Differential Scanning Calorimetry

Measurements by differential scanning calorimetry were carried out using the Linseis Chip 100 calorimeter. Test samples weighing 2 mg were placed in aluminum crucibles with a capacity of 20 µL. They were heated in the temperature range from 40 to 300 °C under a nitrogen atmosphere. The heating speed was 10 degrees/min. The measurements of pure ezetimibe, pure cyclodextrin, and the obtained drug-cyclodextrin complex were performed using this method.

#### 3.2.3. Phase Solubility Study

**Figure 16:**
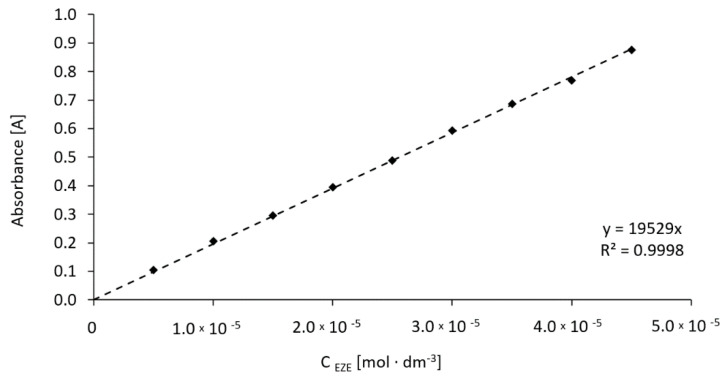
Calibration curve of ezetimibe for λ = 232 nm.

Spectrophotometric studies were carried out using the single-beam UV-Vis spectrophotometer SPECORD 50 (Analytik Jena, Jena, Germany). The maximum absorption of the ethanolic solutions of ezetimibe was marked at a wavelength of 232 nm. The calibration curve of the drug was set for the concentration range from 5.0 × 10^−6^ M to 4.5 × 10^−5^ M. The determined molar extinction coefficient of ezetimibe was 19,529 M^−1^cm^−1^ (Figure 16). The drug solubility in the water, established experimentally, was 8.7 × 10^−7^ M. To investigate the complex formation effect on the ezetimibe solubility in the water, three measurement series were prepared. The excess of ezetimibe was placed in Eppendorf tubes containing αCD, βCD, HPβCD, and SBEβCD solutions. The concentrations of the solutions were in the range of 1 to 90 mM for αCD, 1 to 15 mM for βCD, 1 to 90 mM for HPβCD, and 1 to 90 mM for SBEβCD. The tubes were stored for 7 days at 298.15 K until equilibrium. After this time, the solutions were centrifuged using a Microcentrifuge MPW-55 at 13,000 rpm for 5 min. The pellucid solutions were collected from above the sediment. Afterwards, they were properly diluted so that the UV-Vis spectrum was within the spectrophotometer operating range.

#### 3.2.4. FT-IR

Spectrophotometric measurements, ranging from 450 to 4000 cm^−1^ at room temperature, were carried out using a Nicolet iS5 Thermo Fisher Scientific (Waltham, MI, USA) spectrophotometer. The measurements were performed using the transmission method, with the use of potassium bromide as a carrier for all tested samples. For each series, the measurement contained spectra of pure ezetimibe, pure cyclodextrin, and drug-CD complex. The complexes were made using the co-evaporation method, the same way as the differential scanning calorimetry measurements. The samples, with a 200-fold excess of KBr on a mass ratio, were prepared by thoroughly mixing and grinding them with a mortar.

#### 3.2.5. ^1^HNMR

^1^HNMR spectra of deuterated dimethyl sulfoxide (DMSO-d6) with ezetimibe (EZE) with a concentration of 0.42 mM, β-cyclodextrin (β-CD) with a concentration of 0.42 mM, and the complex of β-cyclodextrin and ezetimibe with a concentration of 0.42 mM, were made with the use of a Bruker Avance III 600 MHz NMR spectrometer at temperature 25 °C

#### 3.2.6. MALDI TOF MS

MS spectra were performed for ezetimibe, β-cyclodextrin, and the complex ezetimibe-β-cyclodextrin in a ratio of 1:1. The samples were dissolved in methanol (1 mg/mL) with alfa-cyano-4-hydroxycinnamic acid (CHCA) as a matrix (10 mg/mL H_2_O/ACN 1:1). The measurement was accomplished using a mass spectrometer Axima Performance MALDI-TOF/TOF (Shimadzu, Kyoto, Japan).

#### 3.2.7. Hirshfeld Surface Analysis

The Hirshfeld surfaces for ezetimibe and βCD were generated using Crystal Explorer 17 [36], based on the X-ray studies results, obtained from CSD (Cambridge Structural Database, version 5.41, 2019 release, Cambridge, United Kingdom) [48]. The bond lengths of hydrogen atoms were normalized to standard neutron values (C–H = 1.083 Å, O–H = 0.983 Å, N–H = 1.009 Å). The normalized contact distance (d*_norm_*) based on both d*_e_* (the distance from a point on the surface to the nearest atom outside the surface) and d*_i_* (the distance from a point on the surface to the nearest atom inside the surface) and van der Waals radii of the atom, given by the Equation (2):

(2) $$d_{norm}=\frac{d_{i}+r_{i}^{vdW}}{r_{i}^{vdW}}+\frac{d_{e}+r_{e}^{vdW}}{r_{e}^{vdW}}$$

enables the identification of the regions of particular importance to intermolecular interactions [49]. The value of d*_norm_* is negative (red color) or positive (blue color) when intermolecular contacts are shorter or longer than van der Waals separations, respectively.

#### 3.2.8. Biochemical Analysis

Mitochondria were isolated from the rat liver by the method of differential centrifugation [50]. We used the isolation medium containing 0.25 M sucrose, 0.02 M Tris-HCl, 0.001 M EDTA, pH 7.2, 4 °C. The mitochondrial pellet was resuspended in the buffer to a protein concentration of 35–40 mg/mL.

Swelling of mitochondria was measured from the changes in the absorbance of mitochondrial suspension at 520 nm and 25 °C using a buffer containing 0.25 M sucrose, 0.02 M Tris-HCl and 0.001 M KH2PO4, pH 7.2 [51]. Isolated mitochondria (0.5 mg of protein/mL) were added to the medium containing respiratory substrate (5 mM succinate). After 30 s of incubation, βCD/ezetimibe/complex ezetimibe—βCD (10–50 µM) were added and the rate (ΔD^520^/min) of the termination phase of swelling was measured.

Mitochondrial membrane potential was detected with a Solar CM 2203 spectrofluorimeter (Belarus), using the fluorescent dye safranin O (8 µM) at λ_ex_/λ_em_ 495/586 nm [52,53] and the buffer containing 0.05 M sucrose, 0.01 M Tris-HCl, 0.125 M KCl, 2.5 mM.

KH_2_PO_4_, 5 mM MgSO4, pH 7.5. Measurement of potential was performed at 27 °C using 5 mM succinate as substrate. Isolated mitochondria were added to the media at a concentration of 0.3 mg of protein/mL and after 300 s βCD/ezetimibe/complex ezetimibe −βCD (10–50 µM) were added. The positively charged dye accumulated in mitochondria depending on their potential, with the intramitochondrial dye accumulation resulting in fluorescence quenching. The mitochondrial membrane potential values (mV) were determined using a calibration plot which represented the dependence of safranine O fluorescence intensity on the mitochondrial membrane potential value, calculated according to the Nernst equation: where the [K+]in is the intramitochondrial potassium concentration (120 mM) and the [K+]out is the extramitochondrial potassium concentration in the medium that varies from 0 to 20 mM [52,53]. For calibration, the membrane potential values were changed by the varying extramitochondrial potassium concentration in the medium in the presence of ionophore valinomycin (0.28 μM). Complete depolarization of mitochondria to calibrate the dye fluorescence was ΔΨ = 60log [K+]out/[K+]in (mV), achieved by addition of FCCP (0.5 µM). To express the dependence of mitochondrial potential on calcium concentration, we used the value of potential change per min (mV/min).

## 4. Conclusions

Based on the presented studies, it can be concluded that ezetimibe inclusion complexes with α-cyclodextrin, β-cyclodextrin, and its derivatives, were formed in a molar ratio of 1:1. ITC measurements of all ezetimibe-cyclodextrin complexes indicate spontaneity (ΔG < 0) and the spontaneous nature of the ligand-receptor interaction process. The formation of complexes of this drug with cyclodextrins is controlled by enthalpy (Δ*H* < 0) and entropic (ΔS > 0) processes. The complex stability constants, which were determined by the calorimetric titration method, with values greater than 1000 (*K* > 1000 dm^3^ mol^−1^) indicates the formation of stable inclusion complexes.

The formation of the complexes is also confirmed by spectrophotometric. In all studied cases, the linear growth of ezetimibe, included in cyclodextrin molecule, was observed. The stability constant value of the αCD complex, established by the Higuchi–Connors solubility method (125.20 ± 6.74 dm^3^ mol^−1^), was lower than the results obtained via the ITC method (815 ± 106 dm^3^ mol^−1^). The most probable reason for the achieved result is the fact, that UV measurements were performed using the static method (Higuchi–Connor’s method), while the calorimetric titration is classified as a dynamic method that measures real-time thermal effects [54]. The stability constants of the αCD-EZE complex, established by both methods, take lower values in comparison to βCD and its derivatives. This most likely indicates a worse spatial fit of the drug molecule to the hydrophobic interior of α-cyclodextrin.

Based on the registered DSC thermograms (Figure 5) of ezetimibe complexed with αCD, βCD, HβCD, and SBE-βCD, the disappearance of characteristic peak at around 162 degrees Celsius (corresponding to the decomposition of the investigated drug) can be observed. DSC measurements clearly show that the decomposition temperature of ezetimibe, which is complexed with cyclodextrins, significantly shifts towards higher temperatures. The formation of ezetimibe complexes with cyclodextrins improves the thermostability of this drug.

The FT-IR spectra of the complexes show bands, at wavelengths of 1224.0 cm^−1^ from the C-F group and bands at 1509.7 cm^−1^ from aromatic C = C, which come from ezetimibe. The 1730.6 cm^−1^ band, derived from the C = O bond, disappears in all analyzed complexes. The disappearance of this band is caused by the shielding of the drug molecule by the cyclodextrin molecule.

MALDI-TOF-MS and ^1^HNMR results confirm the formation of the complex between ezetimibe and β-cyclodextrin.

The biological studies could suggest that the studied complex induced pore Ca^2+^ formation independently due to direct interactions with membrane proteins participating in pore formation.

As a result of this study, we managed to confirm the formation of a complex of ezetimibe with cyclodextrins, define the thermodynamics and stoichiometry of the processes taking place. We also proved the increase of solubility of the analyzed drug caused by the presence of cyclodextrin molecules and we determined how the complexation process affects the thermal properties of ezetimibe.

## Figures and Tables

**Table 1:** Thermodynamic parameters of ezetimibe inclusion complexes with selected cyclodextrins.

	αCD	βCD	HPβCD
*N*	0.85 ± 0.22	1.08 ± 0.06	0.82 ± 0.25
*K* [dm^3^ mol^−1^]	815 ± 106	1710 ± 122	2940 ± 266
Δ*H* [J mol^−1^]	−267.83 ± 80.39	−641.42 ± 41.03	−710.92 ± 53.17
Δ*S* [J mol^−1^ K^−1^]	54.85	59.87	64.06
Δ*G* [kJ mol^−1^]	−16.24	−18.03	−19.35

**Table 2:** Experimentally determined increase in ezetimibe solubility for selected cyclodextrins up to 15 and 90 mM and stability constants calculated by the Higuchi–Connors method.

	αCD	βCD	HPβCD	SBEβCD
Increase of solubility 0–15 mM	4.09 ± 0.27	21.20 ± 1.21	21.22 ± 0.68	26.12 ± 0.71
Increase of solubility 0–90 mM	13.07± 0.60		142.41 ± 2.88	214.19 ± 5.25
*K* [dm^3^ mol^−1^]	125.20 ± 6.74	1324.37 ± 130.70	1580.06 ± 42.27	2346.6 ± 85.46

## Data Availability

The data presented in this study are available on request from the corresponding author.
